# Novelty upon novelty visualized by rotational scanning electron micrographs (rSEM): Laboulbeniales on the millipede order Chordeumatida

**DOI:** 10.1371/journal.pone.0206900

**Published:** 2018-11-28

**Authors:** Ana Sofia P. S. Reboleira, Henrik Enghoff, Sergi Santamaria

**Affiliations:** 1 Natural History Museum of Denmark, University of Copenhagen, Universitetsparken 15, DK København Ø, Denmark; 2 Unitat de Botànica, Departament de Biologia Animal, de Biologia Vegetal i d’Ecologia, Facultat de Biociències, Universitat Autònoma de Barcelona, 08193-Bellaterra, Barcelona, Spain; Pontificia Universidade Catolica do Rio Grande do Sul, BRAZIL

## Abstract

Laboulbeniales are highly specific ectoparasitic fungi of arthropods (insects, millipedes, and arachnids). The first Laboulbeniales parasitizing the millipede order Chordeumatida (Diplopoda) were discovered and described as a new dioecious genus of Laboulbeniales, *Thaxterimyces*, to accommodate the new species *T*. *baliensis*. Also the millipede host is a new species and is described as *Metopidiothrix sheari*. This is the first time Laboulbeniales fungus and its millipede host are described as new together. Males of *Metopidiothrix* have the most extensive secondary sexual modifications in the entire class Diplopoda. Although nothing is known about the function of these modifications, the unique pattern of Laboulbeniales infection in the new millipede species is obviously related to host sexual behavior. Rotational Scanning Electron Micrographs (rSEM) are used to create a 3D comprehensive model to examine the fungal-host interaction, a more advanced visualization of the ectoparasitic fungus on its host. Laboulbeniales diversity on millipedes is still understudied, and a consistent effort is needed to unveil and understand the extent and diversity of this biological interaction. Due to their minute size and apparently non-detrimental effect on their hosts, Laboulbeniales in general have been largely ignored by mycologists and neglected by generations of entomologists. As a result a significant component of global biodiversity has been strongly underestimated, and a wealth of new discoveries is still to be made both in the field and in existing museum collections.

## Introduction

Laboulbeniales constitute an exceptional order of ascomycetous fungi. All are known to be obligate ectoparasites growing only on the surface of living arthropods [1]. Known from arachnids, millipedes and insects, these fungi are characterized by a reduced and compact hyphal system, called thallus, comprising a receptacle bearing one or more perithecia and/or sterile and male appendages [2]. Laboulbeniales have no known anamorphic phase, i.e., the thalli always develops directly from an ascospore released from a perithecium. The thalli are strongly attached to the host’s integument by a foot, which in some taxa penetrates into the body cavity of the host with rhizoidal hyphae and haustoria. The occurrence of Laboulbeniales follows two major tendencies: host specificity and restriction to grow on specific parts of the host body [3–4]. Due to the latter tendency, Laboulbeniales are characterized as “behaviorally transmitted” ectoparasites [5].

The absolute dependence on a host makes Laboulbeniales ideal subjects for the study of co-evolution, with a remarkable unexplored potential to be applied to a wide range of biological questions, including tracking migratory behavior of hosts [6–7].

Millipedes as hosts for Laboulbeniales have been neglected, compared to insects, especially beetles [8], and they were considered rare on millipedes [9, 10]. However, recent studies have revealed a significant diversity and knowledge on their biology has increased considerably [10–11–12–13–14]. For example, based almost exclusively on the study of a single museum collection, nine new species of the genus *Rickia* were described from millipedes [11]. This clear evidence of the hidden biodiversity in collections underlines a huge potential for future field assessments.

Among samples deposited in the Natural History Museum of Denmark, a new genus and species of Laboulbeniales was found attached to a new species of millipede host–the first Laboulbeniales host of the order Chordeumatida.

Chordeumatidan millipedes are mostly distributed in temperate and alpine climates with only a few genera occurring in tropical areas. Among their tropical representatives, the family Metopidiothrichidae is the most diverse. It is characterized by the synapomorphy of the enlarged and partially sclerotized coxal glands in the 10^th^ pair of male legs [15] and comprises seven genera: *Nipponothrix* Shear & Tanabe, 1994, *Australeuma* Golovatch, 1984, *Nesiothrix* Shear & Mesibov, 1997, *Reginaterreuma* Mauriès, 1987, *Neocambriosoma* Mauriès, 1987, *Schedotrigona* Silvestri, 1903 and *Metopidiothrix* Attems, 1907, distributed in southeastern Asia, tropical and temperate Australia, New Zealand and Japan. The genus *Metopidiothrix*, revised by Shear [15], has 38 described species and is remarkable because males possess the most extensive secondary sexual modifications in all millipedes.

## Materials and methods

### Laboratory procedures

For scanning electronic microscopy, specimens were critical point dried in a Tousimis Autosamdi 815, serie A. Others were transferred to 96% ethanol, then to acetone, air-dried, mounted on aluminium stubs, coated with platinum/palladium and studied in a JEOL JSM-6335F scanning electron microscope.

The fungi was detached from the host by micromanipulation and mounted on a permanent slide with lactophenol [11]. Photomicrographs of the fungus were made with a Jenoptik ProgRes 10 Plus digital camera on a Leica DMR microscope equipped with differential interference contrast optics (DIC). Images were processed with Photoshop CS5 software and Dpx View Pro for its included feature of extended focus function.

A critical point dried infected leg of the host was used to create a 3D model, following the procedure described by Cheung et al. [16].

Material is deposited in the Natural History Museum of Denmark, University of Copenhagen (ZMUC, C-F) and in the Autonomous University of Barcelona, Spain (BCB).

### Nomenclatural acts

The electronic edition of this article conforms to the requirements of the amended International Code of Zoological Nomenclature, and hence the new names contained herein are available under that Code from the electronic edition of this article. This published work and the nomenclatural acts it contains have been registered in ZooBank, the online registration system for the ICZN. The ZooBank LSIDs (Life Science Identifiers) can be resolved and the associated information viewed through any standard web browser by appending the LSID to the prefix “http://zoobank.org/”. The LSID for this publication is: urn:lsid:zoobank.org:pub:6F26E6F5-2C4A-4D7D-AA09-902523365AF0. The electronic edition of this work was published in a journal with an ISSN, and has been archived and is available from the following digital repositories: PubMed Central, LOCKSS.

The electronic version of this article in Portable Document Format (PDF) in a work with an ISSN or ISBN will represent a published work according to the International Code of Nomenclature for algae, fungi, and plants, and hence the new names contained in the electronic publication of a PLOS ONE article are effectively published under that Code from the electronic edition alone, so there is no longer any need to provide printed copies.

In addition, new names contained in this work have been submitted to MycoBank from where they will be made available to the Global Names Index. The unique MycoBank number can be resolved and the associated information viewed through any standard web browser by appending the MycoBank number contained in this publication to the prefix http://www.mycobank.org/MB/. The online version of this work is archived and available from the following digital repositories: PubMed Central, and LOCKSS.

## Results

### Taxonomic treatment of the host

Suborder Heterochordeumatidea Shear, 2000

Superfamily Heterochordeumatoidea Pocock, 1894

Family Metopidiotrichidae Attems, 1907

Subfamily Metopidiotrichinae Attems, 1907

Genus *Metopidiothrix* Attems, 1907: 125

## *Metopidiothrix sheari* Reboleira & Enghoff, new species

**Fig 1:**
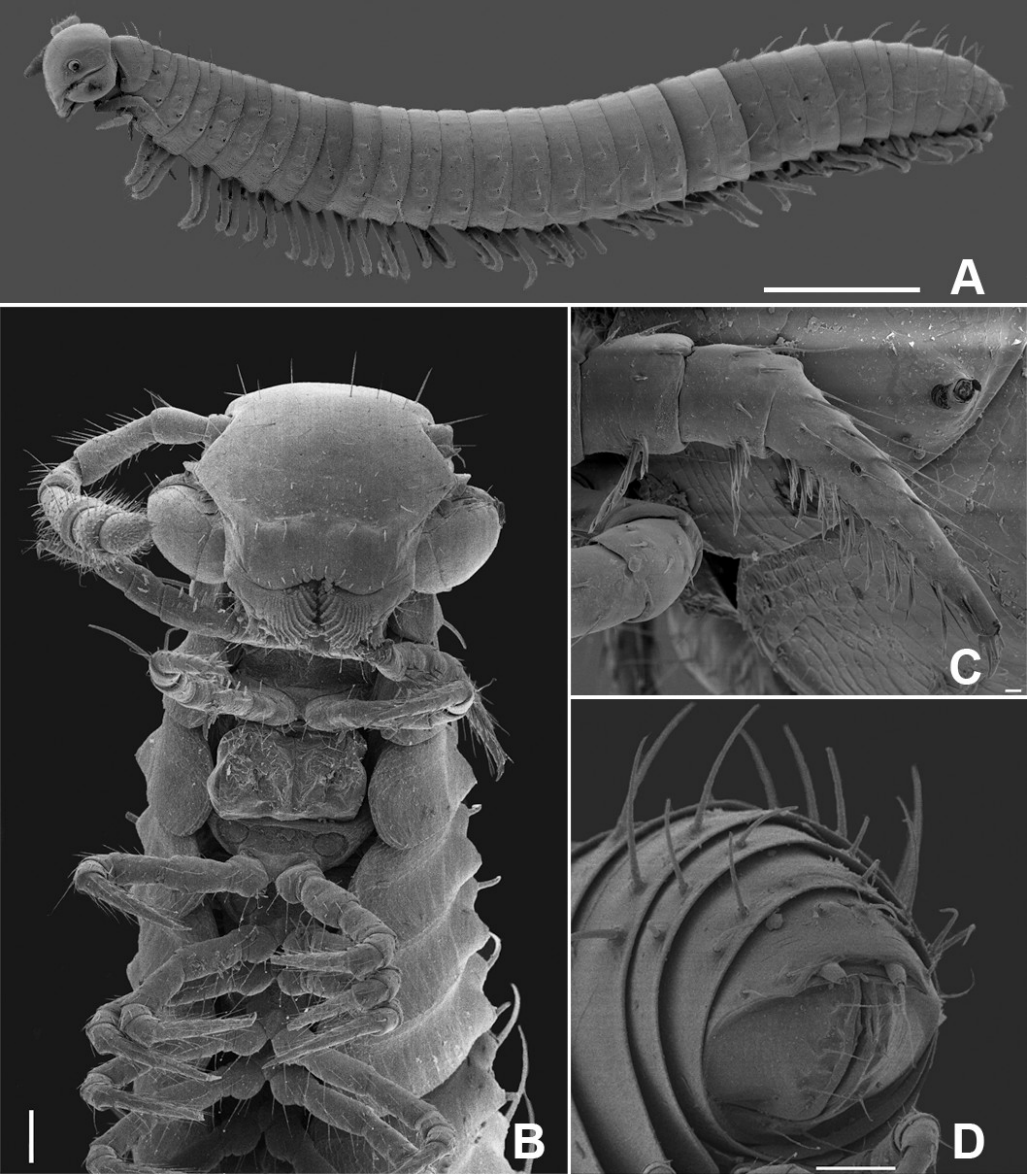
Female of *Metopidiothrix sheari*, scanning electron micrographs. (A) habitus. (B) ventral view of anterior end. (C) knifelike setae on first pair of legs. (D) telson with spinnerets. Scale bars: A, 1 mm; B, D, 100 μm and C, 10 μm.

**Fig 2:**
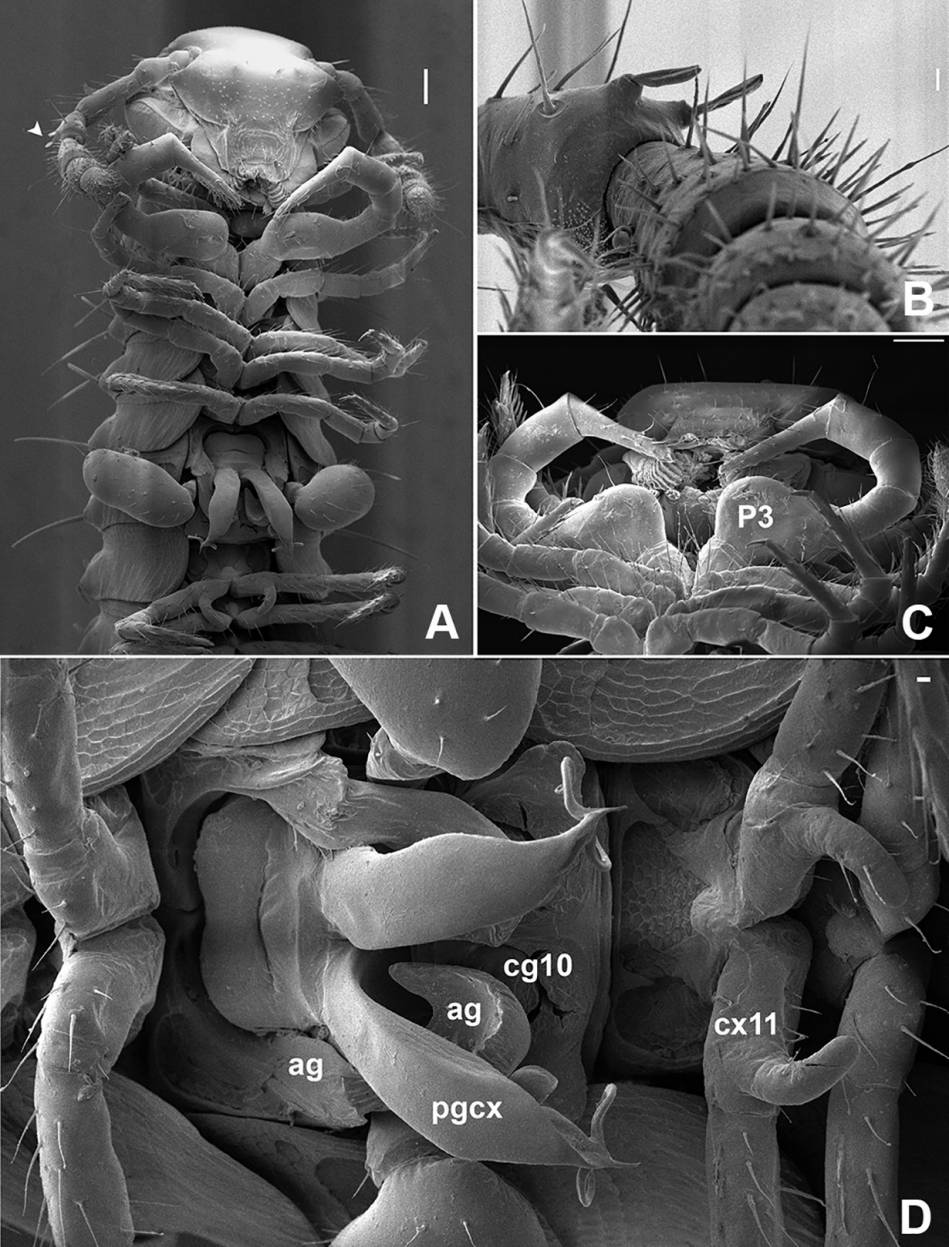
Male of *Metopidiothrix sheari*, scanning electron micrographs. (A) ventral view of anterior end. (B) Modified sensilla in the antenna. (C) Leg-pair 3. (D) ventral view of leg-pairs 7 to 12 *in situ*. P3, leg 3. *ag*, anterior gonopod; *pgcx*, posterior gonopod colpocoxite; *cg10*, coxal gland of leg 10; *cx11*, coxa of leg 11. Scale bars: A, C, 100 μm and B, D, 10 μm.

**Fig 3:**
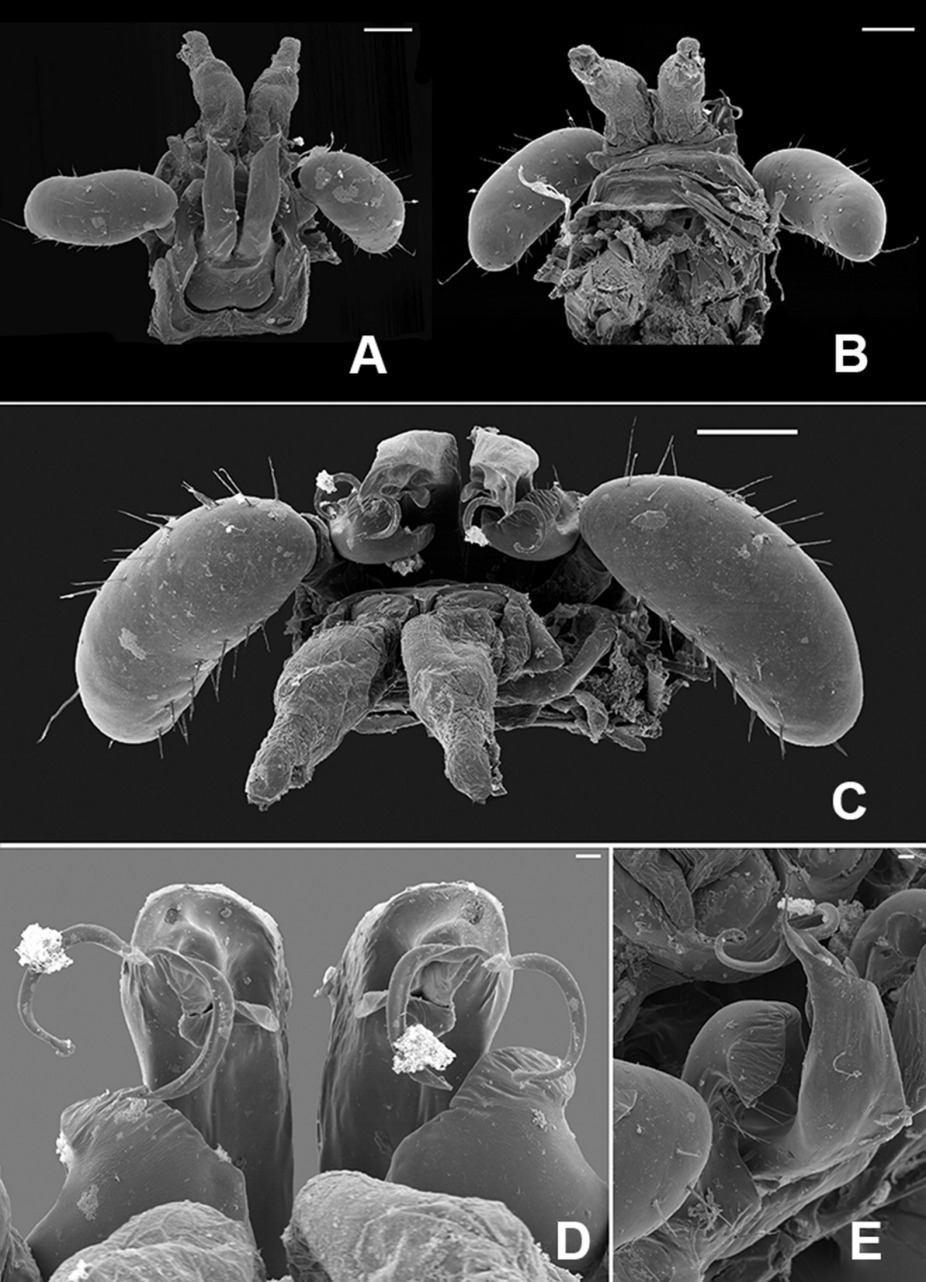
Gonopods of *Metopidiothrix sheari*, scanning electron micrographs. (A) anterior view. (B) posterior view. (C) ventral view. (D) detail of the tip of posterior gonopod. (E) lateral view. *ags*, anterior gonopod sternum; *ag*, anterior gonopod; cg10, coxal gland P10; t9, telopodite of posterior gonopod; pgc, posterior gonopod coxa; *pgcx*, posterior gonopod colpocoxite; *t10*, telopodite 10. Scale bars: A, B and C, 100 μm; D and E, 10 μm.

[urn:lsid:zoobank.org:act:409841BC-CF35-4073-8EE5-588507B95D20] (Figs 1–3)

## Etymology

The species is named after our colleague William Shear, in recognition of his contribution to the study of the genus *Metopidiothrix*.

## Type material

**Holotype:** INDONESIA: Bali Island, Penulisan, Pura Tegeh Kuripan, 17.vii.2014, S 8°12’29”, E 115°19’30”, 1720 m asl, J. Pedersen & A. Schomann leg. (male, ZMUC00039880). **Paratypes:** Same data as holotype (12 females, one female coated for SEM, 17 males, one male coated for SEM, ZMUC00047108).

## Diagnosis

Easily distinguished from any other species of *Metopidiothrix* by the combination of the unique cyphopod shape, the flattened male head, the enlargement of the third pair of male legs, the distinctive anterior and posterior gonopods, including two slender processes on apex of the latter and extremely reduced glabrous telopodite podomere on leg 10 in males (cf. [15]).

## Description

Body pigmented dark grey, head paler, legs yellowish. Both sexes with 31 body segments (plus telson). Medium size, length: 6.6–7.5 mm in males and 7.3–8.1 mm in females, width 0.63–0.75 mm in males and 0.75 mm in females. Antenna long, reaching 3^rd^ body ring, eye with 12 pigmented ommatidia. Three macrosetae on each side, each on prominent tubercle, distance between lateral and intermediate setae smaller than distance between intermediate and mesal setae (Fig 1A). First and second pairs of legs with strong, knifelike setae ventrally (Fig 1C). Two spinnerets on the epiproct (Fig 1D).

## Male

Head modified, clypeolabral part flattened, generating angular projection in front of antenna (Fig 2A), labral hook absent. Fourth antennomere with two distal bumps with modified sensilla (Fig 2B). Third pair of legs much enlarged, prefemur with huge ventral, almost hemisphaerical swelling (Fig 2C). Anterior gonopod (ag) typical of genus (Fig 3A–3C), its anterior part curved around posterior gonopod colpocoxite (pgcx), its telopodite erect with five strong setae on mesal surface at about midlength (Fig 3E). Posterior gonopod (pg) directed obliquely caudad, with one pore (probably gland opening) at its tip (p, Fig 3D), subapically with two long, slender, curved processes and a third, much smaller process in between, these processes variable in shape and darker than rest of gonopods; an irregularly folded lamella below and behind apical processes. Tenth pair of legs strongly modified, sternum reduced to oval loop, fused laterally with enlarged coxae, coxae each with a coxal gland (cg10, evaginated in Fig 3) and extremely reduced, glabrous telopodite podomere (t10). Eleventh pair of legs with curved mesal coxal hook (Fig 2D).

## Female

Cyphopods not completely fused, with groove running from anterior to posterior edge and fused in posterior margin (Fig 1B). Syncyphium as in Fig 1B, with five strong setae on anterior margin, nine setae on distal and four setae on proximal anterior area of each valve. Sternum of 3^rd^ pair of legs with sculptured pattern (Fig 1B).

## Habitat

The new species was found in a forest, collected in sifted litter, at 1720 m asl.

## Remarks

The posterior gonopods of *M*. *sheari* resemble the ones of *M*. *hauseri* Mauriès, 1989, distributed in Malaysia, and *M*. *enghoffi* (Mauriès, 1978) distributed in the Bismark Archipelago. These three species differ from all others by having at least one slender process emerging from the posterior surface (not the margin) of the posterior gonopods, but whereas in *M*. *hauseri* and *M*. *enghoffi* there is only one such slender process, there are three in *M*. *sheari*, the anterior gonopods are completely different, and the enlargement of the 3^rd^ pair of legs is also accompanied by two prefemoral lobular expansions in *M*. *hauseri* [15–17]. The anterior gonopods of the new species are particularly similar to those of *M*. *melanocephala* Golovatch, 1984, distributed in Vietnam, from which it can be easily distinguish by the enlargement of the 3^rd^ pair of legs, normal in *M*. *melanocephala* [18].

### Taxonomic treatment of the ectoparasite

Order Laboubeniales Lindau in Engler and Prantl

Suborder Laboulbeniineae Thaxt.

Family Laboulbeniaceae Peyr.

## *Thaxterimyces* Santam., Reboleira & Enghoff, new genus

[urn:lsid:mycobank.org: MB825263]

## Etymology

In honor of Roland Thaxter (1858–1932), devoted to the study of Laboulbeniales during more than 40 years, describing more than one thousand taxa, thus setting the foundations for future studies of these fungi.

## Type species

*Thaxterimyces baliensis* Santam., Reboleira & Enghoff

## Diagnosis

Dioecious. Male thalli consisting of a 3-celled receptacle supporting a terminal simple antheridium, which bears one dorsal spinous process (sx, the remains of the original ascospore apex). Female thalli consisting of a (2-)3-celled primary receptacle giving rise to the primary appendage and one perithecium. Perithecial basal cells (m, n, n’ and VII) not visible because of the disintegration of their cell walls at maturity. Walls of lower perithecial cells not thickened and consequently not distinguishable at maturity. Only the uppermost wall cells visible. At least the row of wall cells bearing the trichogyne with an additional cell. Perithecial apex occupied by a big lip-like cell, which protrudes through a break of the outer wall in this area.

## *Thaxterimyces baliensis* Santam., Reboleira & Enghoff, new species

**Fig 4:**
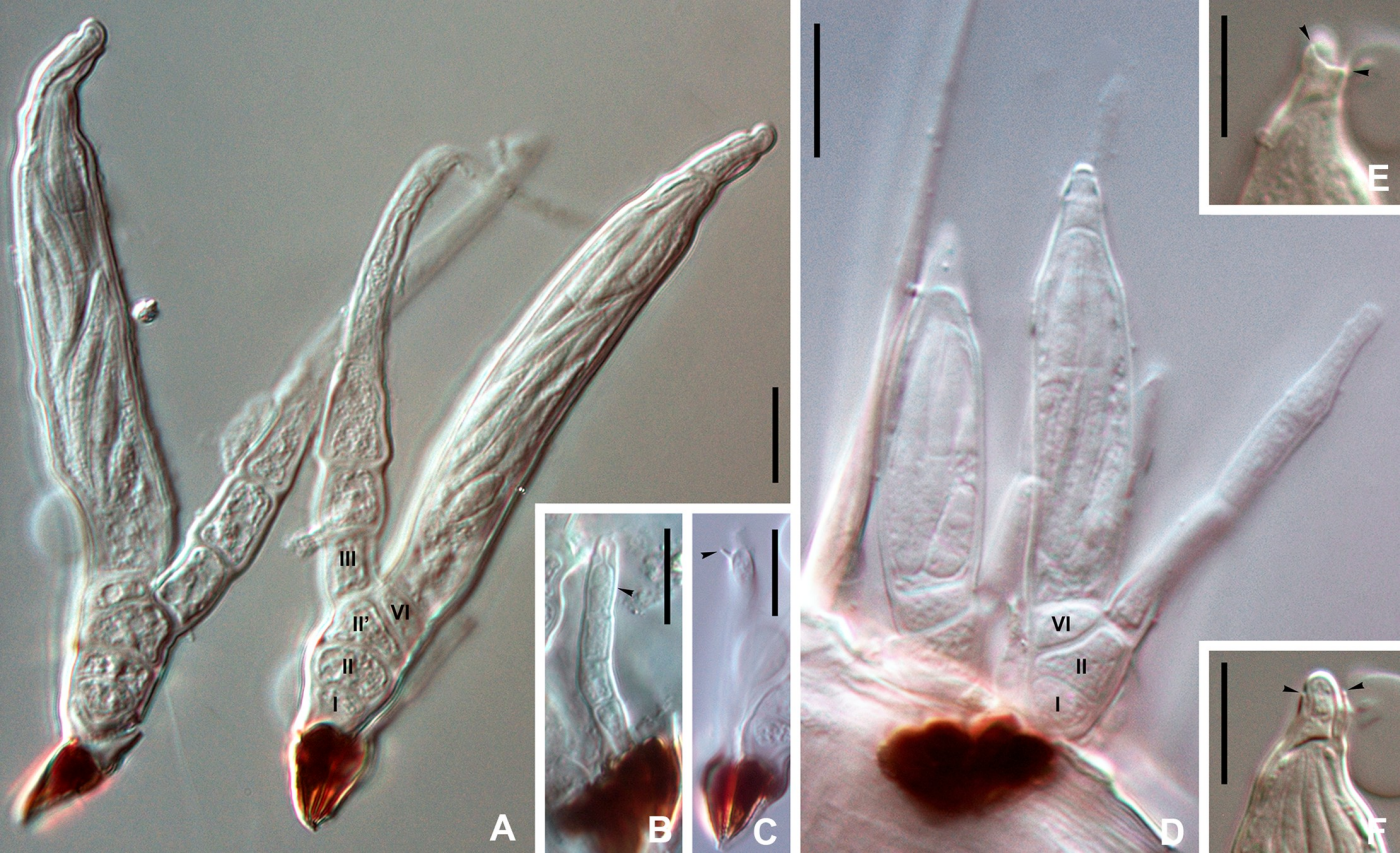
DIC photographs of *Thaxterimyces baliensis*. (A) two females, one with cells I, II, II’, III and VI labelled. (B, C) male thalli at two focusing levels, in B with focused phialid (arrowhead), and in C with focused spinous process (arrowhead). (D) two thallus pairs, males out of focus; thallus at right with cells I, II and VI labelled. E, F. perithecial apex at two focusing levels to highlight the scar of the outer wall break (arrowheads). Scale bars: 10 μm.

**Fig 5:**
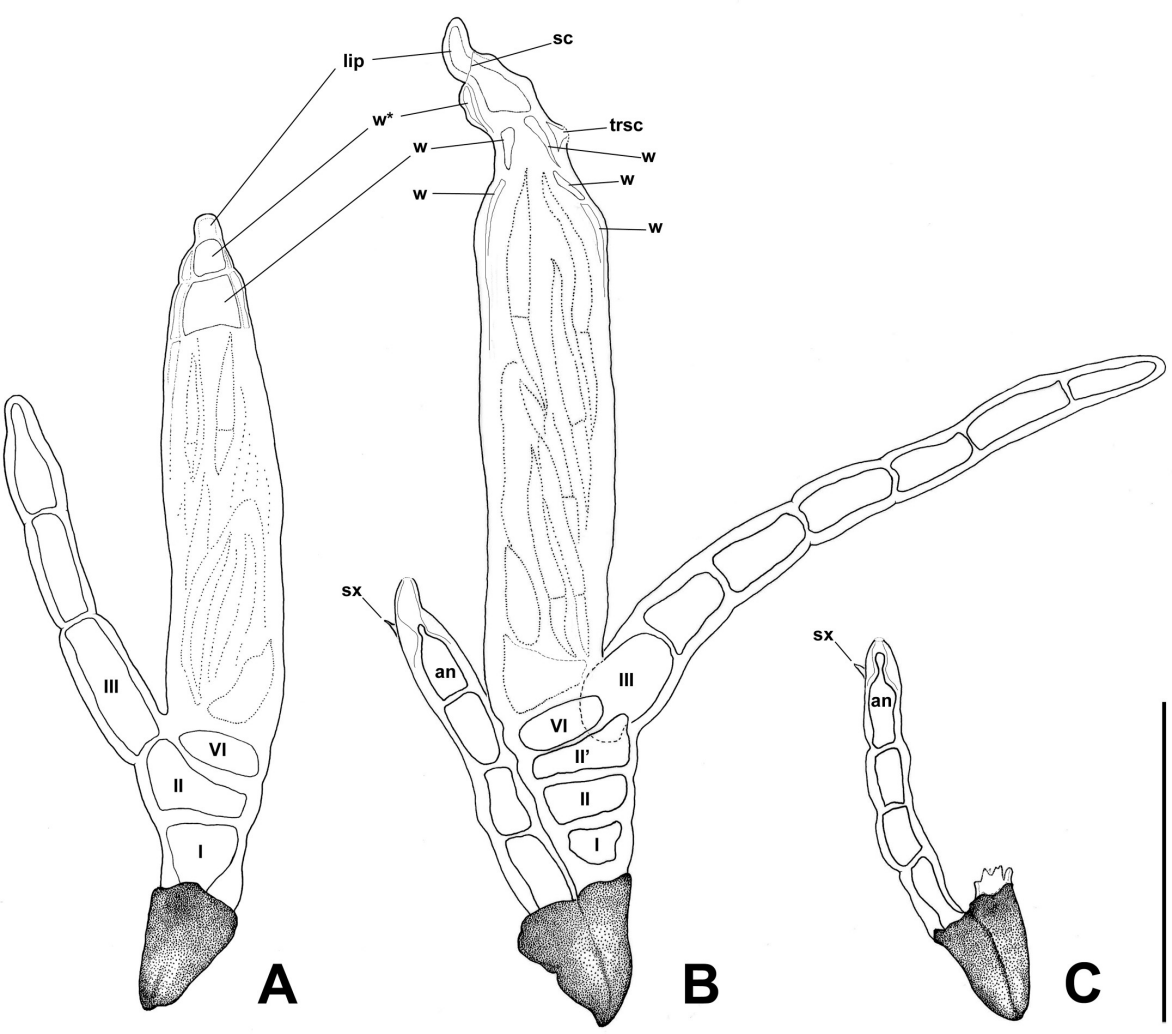
Illustrations of *Thaxterimyces baliensis*. (A) female. (B) paired female and male thalli. (C) male thallus paired with a foot of a broken female thallus. Labels designate main cells and parts: cells I, II, II’, III and VI; perithecial wall cells w, where w*, indicates a characteristic bulging; ‘lip’ points to the lip-like cell; sc, scar of the outer wall break; trsc, trichogyne scar; sx, spinous process; and an, phialide. Scale bar: 50 μm.

**Fig 6:**
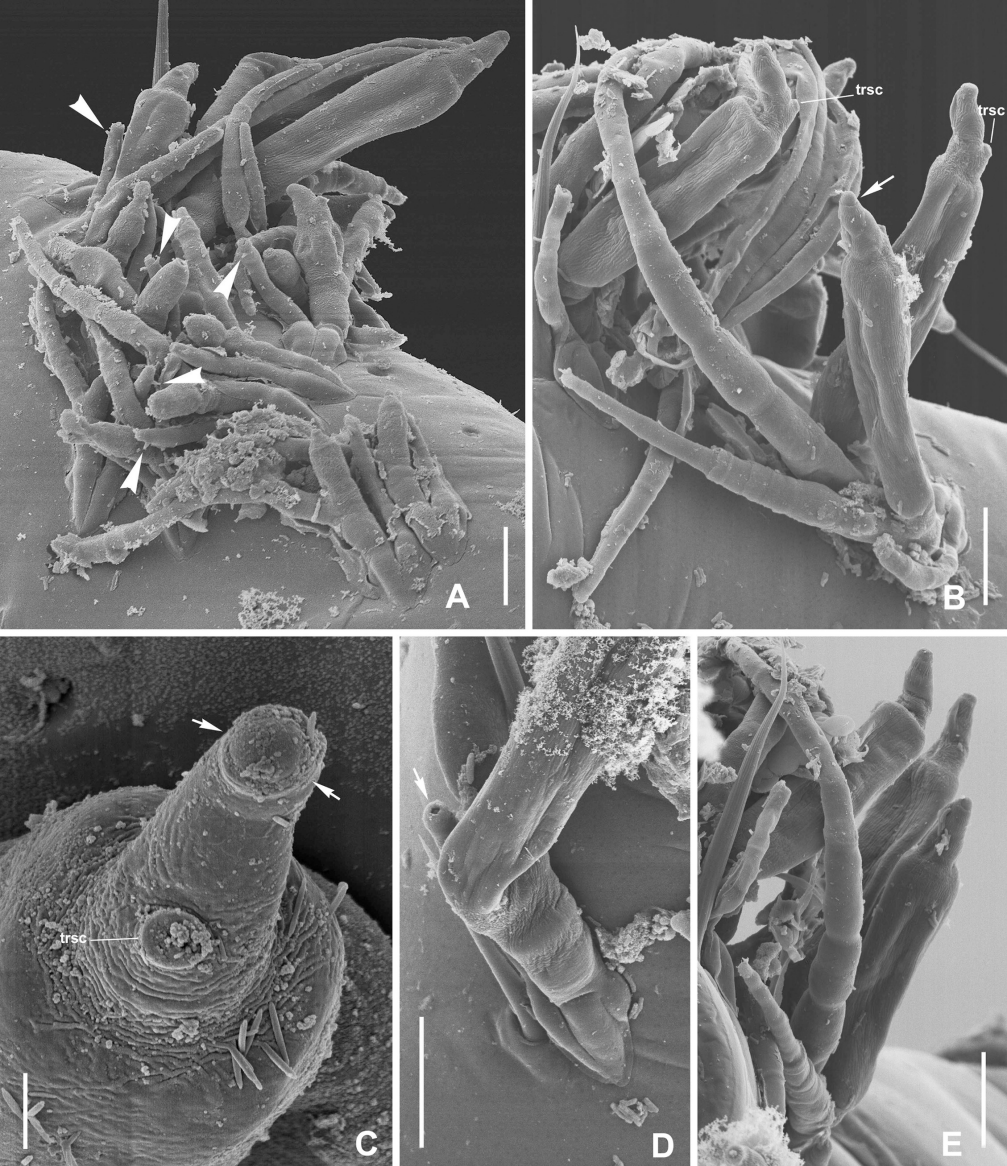
SEM images of *Thaxterimyces baliensis*. (A) whorl of thalli where arrowheads pointing to spinous processes of male thalli (sx). (B) group of thalli showing the trichogyne scars (trsc) and the scar due to the outer wall break near the perithecial apex (arrow). (C) detail of perithecial apex with scar of outer wall break (arrows) and trichogyne scar (trsc). (D) base of paired thalli where arrow points to the antheridium opening. (E) group of thalli. Scale bars: A, B, D, E, 10 μm and C, 2 μm.

[urn:lsid:mycobank.org: MB 825264] (Figs 4–6)

## Etymology

*baliensis*, named after Bali Island, the locality where the hosts have been collected.

## Type material

### Holotype

INDONESIA: Bali Island, Penulisan, Pura Tegeh Kuripan, 17.vii.2014. S 8°12’29”, E 115°19’30”, 1720 m asl, on *Metopidiothrix sheari* sp. nov., J. Pedersen & A. Schomann leg., C-F-92249.

## Other specimens examined

INDONESIA: Bali Island, Penulisan, Pura Tegeh Kuripan, 17.vii.2014. S 8°12’29”, E 115°19’30”, 1720 m asl, on *Metopidiothrix sheari* sp. nov., J. Pedersen & A. Schomann leg., BCB-SS·E581a-c, e (ISOTYPES); BCB-SS·E603af.

## Diagnosis

Same as for genus.

## Description

Dioecious. Male and female thalli hyaline, paired and attached by their respective darkened feet. Male with three almost identical receptacular cells, each about 1.5 times longer than broad, although basal cell slightly longer and constricted below where in contact with darkened foot section. Antheridium of the simple type, spinose, including phialidic cell, which produces spermatia, which extrude through narrow, short opening (Fig 6D arrow). Antheridium vaguely and variably bottle shaped, terminating in inconspicuous, short, or even absent efferent neck. Dorsal surface of antheridium with spinous process (sx, Fig 4C arrowhead, Fig 5BC, Fig 6A arrowheads), remainder of upper original ascospore apex. Female thalli consisting of (2-)3-celled primary receptacle, two-celled condition probably representing young or immature stage. Basal cell (I) as long as broad, strongly constricted below, just above darkened foot, separated from cell II by horizontal septum. Cells II and II’ superimposed, more or less flattened and separated by horizontal septum. Primary appendage unbranched, variably elongated, separated by oblique septum from primary receptacle, consisting of up to seven cells, all longer than broad. Primary septum not clearly distinguished. Tip of appendage rounded. Flattened perithecial stalk-cell (VI) above cell II’ and separated from it by horizontal to slightly oblique septum. Perithecium cylindrical to fusiform, if cylindrical (more typical condition) showing evident parallel sides, at least in lower 3/4. Only upper perithecial wall cells sometimes well distinguished; three of four rows with two short cells, fourth row with three. Row in ventral side including one remarkably bulging uppermost cell (Fig 5AB w*). Row in dorsal side including one additional cell under trichogyne scar (trsc, Fig 5B, Fig 6BC) and one conspicuous large lip-like cell protruding through opening where with careful focusing a scar resulting from break of outer wall in this area can be distinguished (Fig 4EF arrowheads, Fig 5B sc, Fig 6C arrows). Perithecial tip strongly asymmetrical and snout-shaped when seen in lateral view because of protruding, strongly laterally bent lip-like cell. Dorsal surface of perithecial tip uneven above trichogyne scar (Fig 5B). No dimorphism of ascospores observed.

Male 24–39 μm in total length. Antheridium 6–11 × 2–3 μm. Female total length from foot to perithecial apex (40-)56-73(-87) μm. Total length from foot to primary appendage apex (if undamaged) 45–144 μm. Perithecium (27-)36-51(-62) × 8–14 μm.

## Remarks

Initially the new taxon was thought to belong to the genus *Dimeromyces*, but there are several reasons for describing a new genus. *Thaxterimyces* shows affinities with other genera in two groups: the subtribe Euphoriomycetinae in tribe Euphoriomyceteae, and the tribe Dimorphomyceteae, but it cannot be placed in any of these. The subtribe Euphoriomycetinae includes five genera: *Euphoriomyces*, *Carpophoromyces*, *Meionomyces*, *Phaulomyces* and *Siemaszkoa*. The tribe Dimorphomyceteae includes five genera: *Dimeromyces*, *Dimorphomyces*, *Nycteromyces*, *Polyandromyces* and *Trenomyces*. Dioecism, as it occurs in *Thaxterimyces*, does not by itself define a genus, although it should be considered in a genus diagnosis. In Euphoriomycetinae dioecism is an unusual character whereas in Dimorphomyceteae it occurs for all taxa.

The perithecial characters of *Thaxterimyces* agree with both groups by the inability to distinguish the lower perithecial wall cells and the perithecial basal cells in mature specimens. This is due to the evanescence or lack of thickening of the walls of these cells during thallus maturation [1].

Two characters are shared by the new genus and Euphoriomycetinae: (i) a well distinguished perithecial stalk-cell (VI) and (ii) the presence of a supplementary cell in one of the vertical rows of cells of the perithecial wall (Fig 5B). The Dimorphomyceteae lack this extra cell, and cell VI remains undistinguishable. In disagreement with these close similarities with Euphoriomyceteae, certain details of both male and female thalli are shared between *Thaxterimyces* and *Dimeromyces*. The genus *Dimeromyces* was described by Thaxter [19], being one of the biggest genera of Laboulbeniales with 96 species according to Tavares [1], 124 names according to Mycobank databases (http://www.mycobank.org), or 106 accepted species according to Species Fungorum databases (http://www.speciesfungorum.org). After reviewing all data that the genus was concluded to include 112 accepted species parasitizing hosts belonging to several unrelated groups (mites, earwigs, grasshoppers, termites, thrips, flies, and beetles). Within such huge diversity of this genus, the specific characters also exhibit a considerable variability.

Male thalli of *Dimeromyces* have been broadly described as a series of superposed cells giving rise to lateral sterile appendages and antheridia in a variable number and of variable complexity. The appendages may be reduced to the primary appendage only, without any secondary appendage. Antheridia are compound, consisting of a stalk-cell, a venter comprising several basal cells, and a row of antheridial cells which discharge the spermatia into a common cavity before they escape through a terminal opening at the tip of a rather long efferent neck. In spite of the defined compound condition of antheridium, in recent literature, the number of antheridia per thallus has been described as one to several. Based on a review of all descriptions of species of *Dimeromyces* it was concluded that the number of cells producing spermatia is reduced to one in only a single taxon parasitizing *Chiliotis* (Coleoptera Cryptophagidae), i.e. *Dimeromyces chiliotis* Thaxt. This species has 4-celled male thalli lacking a primary appendage, like the new species. The presence of a single phialidic cell in the antheridium prompted Thaxter [20] to establish a new genus under the name *Eudimeromyces*. This genus was later synonymised by Tavares [1] because the lateral growth of the receptacle of females it seemed more appropriate to include *D*. *chiliotis* in the genus *Dimeromyces* or at most in a subgenus. Tavares [1] published a photograph of this species in her monograph where a simple antheridium is well distinguished on the male thallus.

The female thalli, however, clarify the generic adscription: in *Dimeromyces* they are comparable to the male thalli but with the antheridia replaced by the perithecia. The receptacle consists of 3–4 superposed cells or much more. The superposition of cells in the receptacle, i.e. horizontal to oblique septa separating them, is a distinguishing character. Secondary appendages may be wholly absent or copiously developed, simple or branched. The number of perithecia varies among species, although the condition of a single perithecium is more common. In the basal area of the perithecia cells become indistinguishable through the absorption of all the septa, even that of the perithecial stalk-cell (VI).

Similarities with Euphoriomycetinae in general or with *Dimeromyces* among the Dimorphomyceteae in particular notwithstanding, the description of a new genus is justified also by an additional character, i.e., the presence of a spine-like process on the dorsal surface of the antheridium in males. As mentioned above, the spinous process (sx, according to the abbreviation proposed by Tavares [1]) represents the remains of the upper original part of the ascospore, which does not broaden during development. This process appears here and there in Laboulbeniales and is an important character for some tribes, e.g., Stigmatomycetineae. Nevertheless, in *Dimeromyces*, the spinous process (sx) has been described only for *D*. *homophoetae* (Thaxter, 1915) and *D*. *amazonicus* (Thaxter, 1920–21) [21–22], but in both these species male thalli bear free primary appendages, a character absent in *Thaxterimyces*. The lack of a free primary appendage in male thalli is another important feature that distinguishes *Thaxteromyces* from *Dimeromyces*.

Perithecial characteristics of *T*. *baliensis* are rather peculiar, especially those seen near or on the apex. The wall cells are hardly visible in this area, and their number for each vertical row is difficult to establish. The dorsal row, where the trichogyne scar is observed, consists of three cells, including the conspicuous lip-like upper cell. This big cell is responsible for the snout-like shape of the perithecial tip and protrudes through a break of an outer wall, which could only be seen by very careful focusing of the microscope at high magnification (Figs 4EF arrowheads, 5B sc, 6BC arrows). This character is similar to that described for some species of *Troglomyces* (Enghoff & Santamaria, 2015) [12]. In the ventral row, only two wall cells can be distinguished, the uppermost characterized by its bulging appearance, besides the lip-like cell, and seen in frontal vision (Fig 5AB w*) or with SEM microscopy. The other two rows are not well distinguished although they are supposed to contain two cells like the ventral row. The lower wall cells are in agreement with those described for Euphoriomycetinae or *Dimeromyces*, i.e., only one very long cell is visible.

## Position and incidence of the fungus on the host

Most of the fungal thalli are distributed on the host’s legs as seen in rSEM in S1 Movie.

All available specimens of *Metopidiothrix sheari* were infected with *Thaxterimyces baliensis*, and the study of the fungal distribution on the host (Table 1) shows a clear pattern, which differs from what is known in other Laboulbeniales on millipedes [10–11–12–13–14].

**Table 1:** Body dimensions of *Metopidiothrix sheari*, and position of the fungus *Thaxteromyces baliensis* on the millipede.

Sex	Length (mm)	Width (mm)	Fungus position
Male (Holotype)	7.5	0.75	1-15^th^ leg-pairs; ventrolateral margin of the 6^th^ pleurotergite
Male	6.5	0.75	1-15^th^ leg-pairs; 2^nd^ right antennomere
Male	6.5	0.75	1-15^th^ leg-pairs; 4^th^ right antennomere
Male	7	0.63	1-15^th^ leg-pairs; ventrolateral margin of the 6^th^ pleurotergite
Male	7	0.63	1-15^th^ leg-pairs; ventrolateral margin of the 6^th^ pleurotergite
Male	6.63	0.63	1-15^th^ leg-pairs ventrolateral margin of the 6^th^ pleurotergite
Male	7.13	0.63	1-15^th^ leg-pairs; ventrolateral margin of the 6^th^ pleurotergite
Male	7.13	0.63	1-15^th^ leg-pairs; ventrolateral margin of the 6^th^ /7^th^ pleurotergite
Male	6.9	0.63	1-15^th^ leg-pairs; ventrolateral margin of the 6^th^ pleurotergite
Male	7.5	0.75	1-15^th^ leg-pairs; ventrolateral margin of the 6^th^ pleurotergite
Male	7.5	0.5	1-15^th^ leg-pairs; ventrolateral margin of the 6^th^ pleurotergite
Male	7.5	0.5	1-15^th^ leg-pairs; dorsal part of the collum; posterior margin of the head; ventrolateral margin of the 6^th^ pleurotergite
Male	7	0.63	1-15^th^ leg-pairs; ventrolateral margin of the 6^th^ pleurotergite
Male	7	0.63	1-15^th^ leg-pairs; ventrolateral margin of the 6^th^ pleurotergite
Male	7	0.5	1-15^th^ leg-pairs; ventrolateral margin of the 6^th^ pleurotergite
Male	6.9	0.62	1-15^th^ leg-pairs; ventrolateral margin of the 6^th^ pleurotergite
Male	7	0.5	1-15^th^ leg-pairs; ventrolateral margin of the 6^th^ pleurotergite
Male	7.5	0.5	1-15^th^ leg-pairs; ventrolateral margin of the 6^th^ pleurotergite
Female	8.1	0.75	1-5^th^ leg-pairs
Female	7.5	0.75	1-5^th^ leg-pairs
Female	7.5	0.75	1-5^th^ leg-pairs
Female	7.5	0.75	1-5^th^ leg-pairs; antennal insertion
Female	7.5	0.75	1-5^th^ leg-pairs
Female	7.5	0.75	1-5^th^ leg-pairs
Female	7.5	0.75	1-5^th^ leg-pairs
Female	7.5	0.75	1-5^th^ leg-pairs
Female	7.5	0.75	1-5^th^ leg-pairs
Female	7.3	0.75	1-5^th^ leg-pairs
Female	7.5	0.75	1-5^th^ leg-pairs; mandibular cardo and stipes

The fungus is mainly distributed on legs 1 to 15 and on the ventrolateral margin of the 6^th^ pleurotergite in males, while it is distributed on legs 1 to 5 in females. Only in highly infected specimens thalli can be found also on the head, the collum and the ventrolateral margin of the 7^th^ male pleurotergite. As it has been established for other orders of millipedes [10–11–12], Laboulbeniales transmission seems also in Chordeumatida to be related to the copulatory behavior of the host.

## Discussion

This report represents the first simultaneous description of an arthropod species together with its associated ectoparasitic fungus of the order Laboulbeniales. Among the 16 orders of the Class Diplopoda [23], six are now known to be parasitized by Laboulbeniales: Sphaerotheriida, Julida, Spirobolida, Spirostreptida, Callipodida [10–11] and Chordeumatida.

Apart from the genus *Rickia*, which is also present on insects and mites [9–11], the other four genera of Laboulbeniales on millipedes are known to be specific to this class: *Troglomyces* Colla, 1932; *Diplopodomyces* W. Rossi & Balazuc, 1977; *Triainomyces* W. Rossi & A. Weir, 1998, and *Thaxterimyces* [9–10–11–12–24].

This new discovery confirms that patterns of position specificity in Laboulbeniales on millipedes are diverse. While in most cases the position pattern clearly indicates transmission related to sexual behavior, as in *Diplopodomyces veneris* Santam., Enghoff & Reboleira, 2014, *Troglomyces bilabiatus* Santam. & Enghoff, 2015, *Troglomyces triandrus* Santam. & Enghoff, 2015, other species, such as *Troglomyces pusillus* Santam. & Enghoff, 2015 or *Rickia appendicifera* Santam., Enghoff & Reboleira, 2016, exhibit a more random pattern, apparently linked with non-sexual behavior of their hosts [10–11–12]. For now the very consistent pattern of Laboulbeniales infection in *Metopidiothrix baliensis*, affecting legs 1 to 15^th^ and ventrolateral margin of the 6^th^ pleurotergite in males, and legs 1 to 5^th^ in females, must be regarded as clear evidence of sexual transmission.

*Metopidiothrix* being the most extreme genus among all millipedes in terms of diversity of sexual modifications, the study of sexual behavior of *Metopidiothrix* species is a tempting challenge, and may also clarify the mechanism of infection. Although”male *Metopidiothrix* have the most extensive suite of secondary sexual modifications yet encountered in millipedes, involving the head, antennae, pregonopodal and postgonopodal legs” [15], absolutely nothing is known about courtship and mating behavior in *Metopidiothrix*, nor in any other genus of the family Metopidiotrichidae. In general, information on courtship and mating in chordeumatidan millipedes is extremely scant. Haacker [25–26] gave information on *Chordeuma sylvestre* C.L. Koch, 1847 (fam. Chordeumatidae), Tadler [27] on *Craspedosoma transsilvanicum* Verhoeff, 1897 (fam. Craspedosomatidae), and Youngsteadt [28] on the genus *Causeyella* Shear, 2003 (fam. Trichopetalidae). In addition, images of copulating chordeumatidans exist for *Craspedosoma rawlinsii* Leach, 1815 (http://www.bmig.org.uk/species/craspedosoma-rawlinsii, accessed 27 April 2017) and *Amplaria muiri* Shear & Krejca (2007) (fam. Striariidae) [29], and also by images of a copulating pair of *Serbosoma lazarevense* (Ceuca, 1964) (fam. Anthroleucosomatidae) (D. Antić, pers. comm.). From this evidence it can be inferred that in addition to various pre-copulatory actions, the final mating position of chordeumatidans is like that of millipedes in general: venter to venter, with the male’s gonopods on body ring 7 positioned opposite the female’s gonopores on ring 3. This circumstantial evidence is in agreement with the general position of thalli of *Thaxterimyces baliensis* on its host, although for example the preference for the ventrolateral margin of the 6^th^ pleurotergite in males remains unexplainable for the time being.

Millipedes are known to have low dispersal abilities, as indicated, e.g., by the very high degree of endemicity even at high taxonomic levels [23]. Their biogeographical history is therefore difficult to trace, but study of their associated parasites [30], including Laboulbeniales [7], may provide an additional source of clues.

## Supporting information

S1 Movie rSEM of a leg of *Metopidiothrix sheari* n. sp. with several thalli of *Thaxterimyces baliensis* n. gen. n. sp. (Fungus artificially coloured in green). (MP4)
